# Prognostic biomarkers in predicting mortality in respiratory patients with ventilator-associated pneumonia

**DOI:** 10.1186/s43168-021-00062-1

**Published:** 2021-03-18

**Authors:** Nermeen A. Abdelaleem, Hoda A. Makhlouf, Eman M. Nagiub, Hassan A. Bayoumi

**Affiliations:** 1 Department of Chest, Assiut University Hospital, Assiut University, Assiut, Egypt; 2 Department of Clinical Pathology, Assiut University Hospital, Assiut University, Assiut, Egypt

**Keywords:** Red cell distribution width (RDW), Ventilator-associated pneumonia (VAP), Mortality, Neutrophil-to-lymphocyte ratio (NLR), Sequential Organ Failure Assessment (SOFA) score, Respiratory, Mortality

## Abstract

**Background:**

Ventilator-associated pneumonia (VAP) is the most common nosocomial infection. Red cell distribution width (RDW) and neutrophil-lymphocyte ratio (NLR) are prognostic factors to mortality in different diseases. The aim of this study is to evaluate prognostic efficiency RDW, NLR, and the Sequential Organ Failure Assessment (SOFA) score for mortality prediction in respiratory patients with VAP.

**Results:**

One hundred thirty-six patients mechanically ventilated and developed VAP were included. Clinical characteristics and SOFA score on the day of admission and at diagnosis of VAP, RDW, and NLR were assessed and correlated to mortality. The average age of patients was 58.80 ± 10.53. These variables had a good diagnostic performance for mortality prediction AUC 0.811 for SOFA at diagnosis of VAP, 0.777 for RDW, 0.728 for NLR, and 0.840 for combined of NLR and RDW. The combination of the three parameters demonstrated excellent diagnostic performance (AUC 0.889). A positive correlation was found between SOFA at diagnosis of VAP and RDW (*r* = 0.446, *P* < 0.000) and with NLR (*r* = 0.220, *P* < 0.010).

**Conclusions:**

NLR and RDW are non-specific inflammatory markers that could be calculated quickly and easily via routine hemogram examination. These markers have comparable prognostic accuracy to severity scores. Consequently, RDW and NLR are simple, yet promising markers for ICU physicians in monitoring the clinical course, assessment of organ dysfunction, and predicting mortality in mechanically ventilated patients. Therefore, this study recommends the use of blood biomarkers with the one of the simplest ICU score (SOFA score) in the rapid diagnosis of critical patients as a daily works in ICU.

## Background

Ventilator-associated pneumonia (VAP) defined as pneumonia developed after endotracheal tube intubation/mechanical ventilation for more than 48 h [1]. It is the commonest nosocomial infection in intensive care units (ICU) [2, 3].

VAP continues to be a leading cause of morbidity and mortality in the nosocomial setting [4–7]. For those patients who are at risk of acquiring VAP, the likelihood of dying is twice as high as that observed in ventilated patients without VAP, with mortality rates ranging from 33 to 50% [4, 8, 9]. The clinical value of scores and biomarkers are limited in diagnosis and prognosis [10].

The Sequential Organ Failure Assessment (SOFA) score is a simple and prognostic score that can calculate both the number and the severity of organ dysfunction in six organ systems (respiratory, coagulator, liver, cardiovascular, renal, and neurologic) [11]. Patients with a higher SOFA score mostly have a higher mortality rate [12].

Red blood cell distribution width (RDW) is a quantitative measure for size variability of circulating erythrocytes detected in a complete blood count (CBC) [13, 14]. RDW calculated by dividing the standard deviation (SD) of the mean corpuscular volume (MCV) by the MCV and multiplying by 100 to yield a percentage value to be on behalf of the RBC size heterogeneity [15]. Also, it is an inexpensive easily reasonable measurement that acts as a prognostic factor in several diseases [15].

Raised RDW associated with worse prognosis in numerous non-infectious [16–21], infectious diseases [22–24], and sepsis and septic shock [25–28]. The definite pathophysiologic mechanism is still uncertain, but systemic factors that affect erythrocyte homeostasis such as inflammation and oxidative stress, essential components in infection cascade, seem to have an important role [29–31].

The neutrophil-to-lymphocyte ratio (NLR) is a systemic inflammation indicato r[32, 33]. NLR is the number of neutrophils divided by the number of lymphocytes. The physiological immune response of leucocytes to stress is characterized by increasing neutrophils and decreasing lymphocytes [34]. Recently, NLR has been shown to be a prognostic marker in various diseases, such as solid tumors [34–36], cardiovascular disease [34, 37], and chronic obstructive pulmonary disease (COPD) [34, 38]. Besides, NLR became an independent indicator of mortality [34, 39, 40]. The NLR proved to be a simple and even better marker in expecting bacteremia than other parameters as C-reactive protein (CRP) level and white blood cell (WBC) count [41, 42].

### The aim of this study

The goal of this study was to evaluate the prognostic efficiency of red blood cell distribution width (RDW), the neutrophil-lymphocyte ratio (NLR), and the Sequential Organ Failure Assessment (SOFA) score for mortality prediction in respiratory patients with VAP.

## Methods

This study was a prospective observational analytic cohort study with no intervention. It was conducted over a period from April 2018 to December 2019 in the respiratory ICU in a tertiary hospital.

Inclusion criteria are as follows:

1. Age > 18 years old
2. Respiratory failure needs mechanical ventilator support > 48 h. VAP was defined as an acute lower respiratory tract infection in mechanically ventilated patient > 48 h with a new or progressing infiltrate on chest radiograph and who met at least two of the following clinical criteria: body temperature > 38 °C or < 36 °C with no other recognized cause, white blood cell count > 10,000/mm^3^ or < 5000/mm^3^, or a macroscopically purulent tracheal aspirate [1, 4].
3. Only the first VAP episode was included.

Exclusion criteria are as follows:

1. Neutropenia (< 500 cells/ml) before the development of VAP.
2. Conditions are known to influence total and differential WBC counts such as chronic inflammatory conditions, hematologic disorders, history of chemotherapy, or radiotherapy within 4 weeks before enrollment.
3. Conditions are known to affect RDW as anemia due to nutritional deficiency (i.e., iron, vitamin B12, and folic acid). RDW tends to be high in nutritional deficiencies [43] as iron (demonstrated as low MCV < 80 fl [44]) and in B12 and folic acid deficiencies (demonstrated as high MCV > 100 fl [44]); those patients were excluded from the study by evaluating their CBC blood indices as MCV and MCH.
4. Patients with HIV/AIDS

For all patients, demographic parameters (age and gender), vital signs, Glasgow Coma Scale (GCS), laboratory results (complete blood counts: WBC count, neutrophil count, lymphocyte count and NLR (absolute neutrophil count divided by absolute lymphocyte count), RDW, serum creatinine, albumin, and arterial blood gas tests) at time of VAP diagnosis. The SOFA score was calculated at admission to the ICU and on the day of the diagnosis of VAP. Pulmonary X-rays were taken. Intensive care unit (ICU) length of stay, duration of mechanical ventilation before developing VAP, and total ICU stays.

For all patients in whom the clinical suspicion of VAP was confirmed, empirical antimicrobial therapy was started on the first day. Antibiotic therapy has been selected by the critical care team. The outcome of interest was in-hospital mortality or survival.

### Laboratory assessment

Hematological parameters were determined by automated CBC analyzer “Cell Dyne Ruby” (Abbott, Diagnostic ®). The RDW values were obtained as part of the CBC results. The normal reference value ranges in our hospital laboratory are for RDW 11.6–14.8% and for NLR between 0.78 and 3.53.

### Statistical analysis

Data were represented as median ± SD or medians and ranges. Categorical variables were compared with the chi-square test or Fisher’s exact test. Comparison of continuous variables between the two groups was performed using the Mann-Whitney *U* test. The area under the ROC curve (AUC) was calculated for each marker, and the standard error and 95% confidence interval (95% CI) were determined. The overall discrimination performance of a given test is measured by calculating the area under the ROC curve (AUC). AUC is a powerful method to summarize the overall diagnostic accuracy of the test. The value of AUC ranges from 0.5 (no discrimination) to 1 (perfect discrimination). In all tests, *P* < 0.05 was considered statistically significant difference. Statistical analysis was performed using the SPSS version 20.0 software package (IBM SPSS, Armonk, NY, USA).

## Results

A total of 136 patients (63.9% men, 36% women) with a mean age (58.80 ± 10.53) were included in the study. The mean length of ICU stay was 15.76 ± 5.72 days, and 54 patients (39.7%) died during hospitalization. Comparative baseline values of demographic, main laboratory findings, and severity of disease between enrolled patients (82 surviving and 54 non-surviving) were listed in Table 1.

**Table 1:** Demographic characteristics, laboratory, and disease severity for the study population

Variable	Total, *n* (136)	Survivor, *n* (82)	Non-survivor, *n* (54)	*P* value
Age (year)	58.80 ± 10.53	57.68 ± 11.30	60.50 ± 9.06	0.127
Sex				
Male	87 (63.9%)	55 (67.1%)	32 (59.3%)	0.368
Female	49 (36%)	27 (32.9%)	22 (40.7%)	
WBC (× 1000/mm^3^)	12.87 ± 4.74	12.73 ± 4.28	13.97 ± 5.41	0.685
Hg (gm/dl)	12.11 ± 2.19	12.51 ± 2.03	11.65 ± 2.22	0.022
PLT (× 1000/mm^3^)	205.27 ± 104.27	210.36 ± 75.36	197.54 ± 137.52	0.419
RDW (%)	14.06 ± 2.83	12.94 ± 1.87	15.75 ± 3.19	0.000
NLR (%)	9.67 ± 4.00	8.51 ± 3.74	11.43 ± 3.76	0.000
Serum urea (mg/dL)	9.75 ± 5.15	6.54 ± 3.29	11.42 ± 5.19	0.008
Serum creatinine(μmol/L)	120.56 ± 70.11	112.94 ± 68.24	132.36 ± 71.97	0.116
Serum albumin (g/L)	24.68 ± 9.90	25.70 ± 10.45	23.11 ± 8.86	0.139
SOFA score at admission	4.25 ± 1.79	3.68 ± 1.39, 3 (2–7)	5.41 ± 1.92, 5 (2–10)	0.000
SOFA score at diagnosis of VAP	5.67 ± 2.38	4.60 ± 1.48, 4 (3–10)	7.30 ± 2.56, 8 (2–11)	0.000
Duration of mechanical ventilation (days)	12.49 ± 4.95	11.12 ± 3.74	14.57 ± 5.81	0.000
Time of occurrence of VAP (days)	6.25 ± 2.58	5.56 ± 2.37	7.17 ± 2.63	0.001
Length of ICU stay (days)	15.76 ± 5.72	13.89 ± 4.53	18.61 ± 6.18	0.000

*WBC* white blood count, *Hg* hemoglobin, *PLT* platelet, *RDW* red cell distribution width, *NLR* neutrophilic lymphocyte ratio, *SOFA* Sequential Organ Failure Assessment Score, *VAP* ventilator-associated pneumonia, *ICU* intensive care unit

The current study demonstrated that, non-survivors were more likely to be elderly with higher WBC and creatinine and lower albumin without statistical significance. Moreover, non-survivors group had significantly higher RDW and NLR than survivors at the time of VAP diagnosis (mean ± SD, 15.75 ± 3.19 versus 12.94 ± 1.87 in survivors for the former and 11.43 ± 3.76 versus 8.51 ± 3.74 in non-survivors for the latter). SOFA score at admission, SOFA at diagnosis of VAP, the duration of mechanical ventilation, time of occurrence of VAP, and total ICU stay were significantly higher in non-survivors group compared to survivors group (*P* < 0.05 for each).

**Fig. 1:**
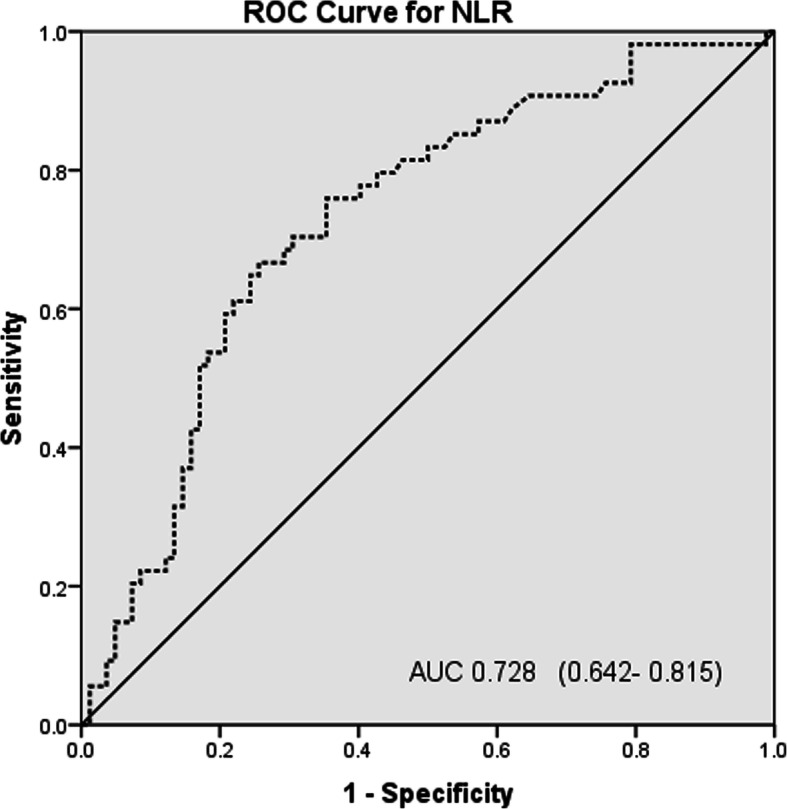
ROC for NLR

**Fig. 2:**
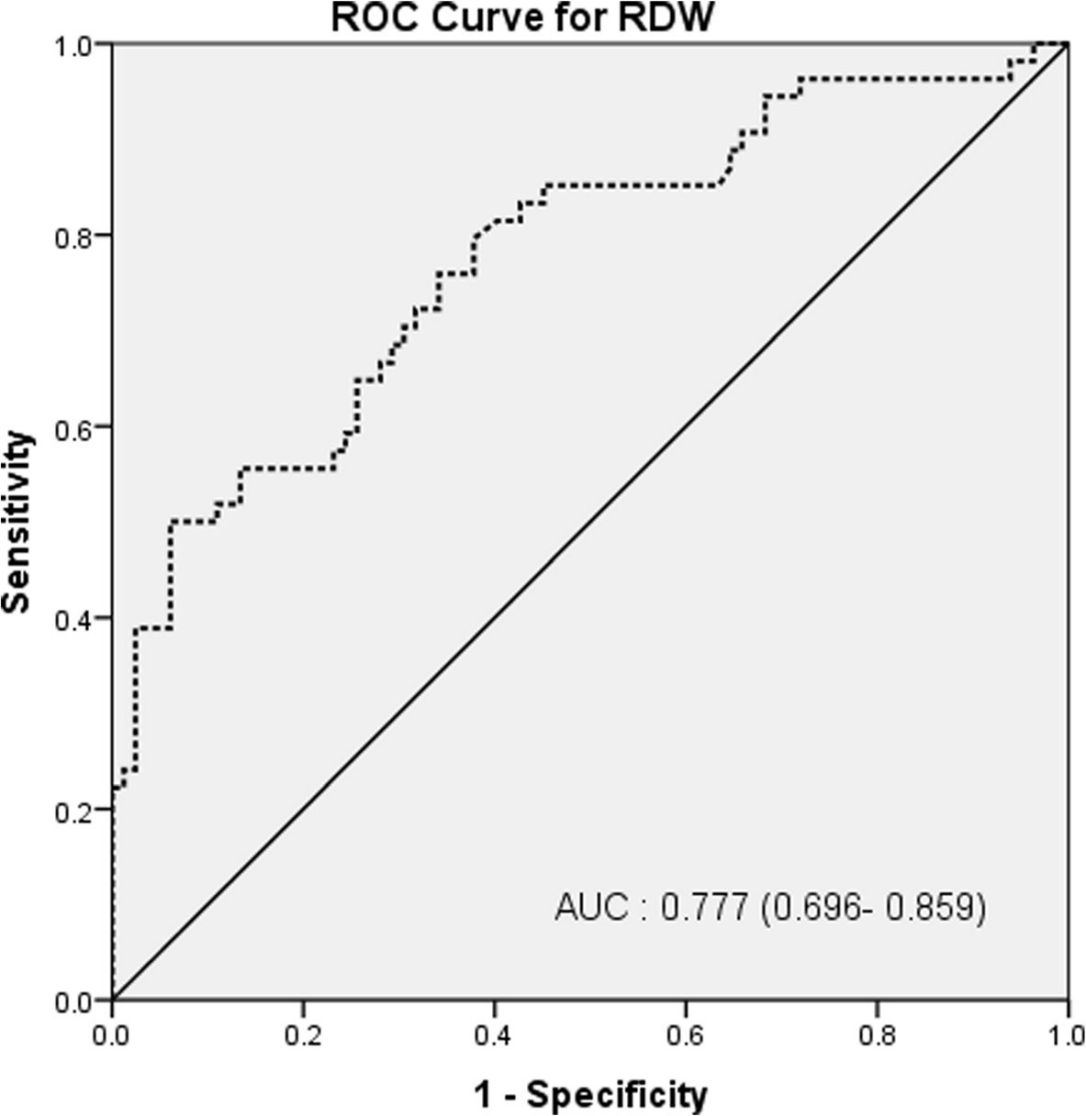
ROC for RDW

**Fig. 3:**
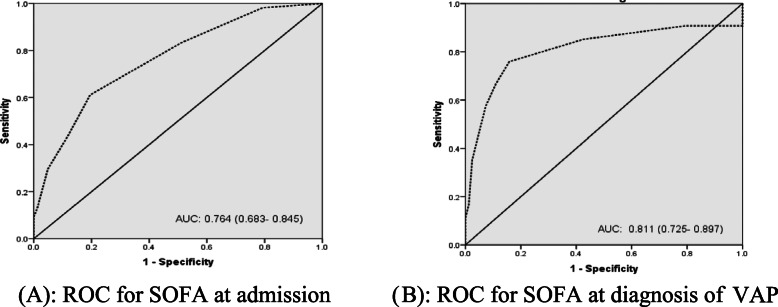
**a** ROC for SOFA at admission. **b** ROC for SOFA at diagnosis of VAP

**Fig. 4:**
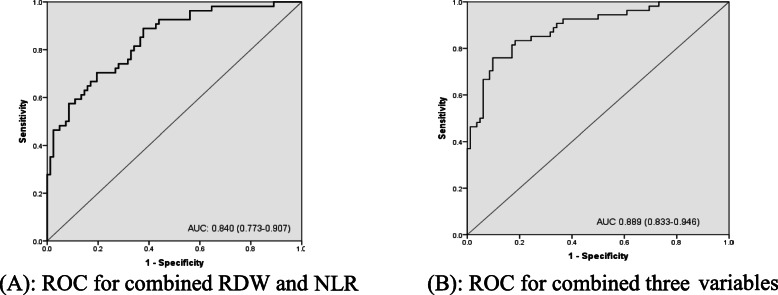
**a** ROC for combined RDW and NLR. **b** ROC for combined three variables

The ROC curve analysis to predict in-hospital mortality showed AUC 0.728 (95% confidence interval [CI] (0.642–0.815)) for NLR (Fig. 1) and 0.777 (95% CI, 0.696–0.859) for RDW (Fig. 2), 0.764 (95% CI, 0.683–0.845) for SOFA at admission (Fig. 3a), 0.811 (95% CI, 0.725–0.897) for SOFA at diagnosis of VAP as shown in (Fig. 3b). The AUCs were significantly increased when RDW was added to NLR 0.840 (95% CI, 0.773–0.907; *P,* 0.000). If the three variables were combined, it gave excellent AUC, 0.889 (95% CI, 0.883–0.946; *P,* 0.000) (Fig. 4a, b).

**Fig. 5:**
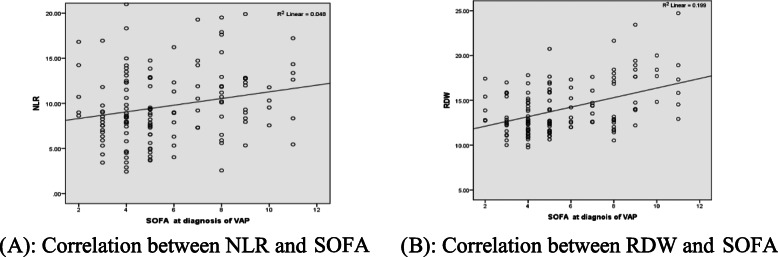
**a** Correlation between NLR and SOFA. **b** Correlation between RDW and SOFA

A positive correlation was found between SOFA score at diagnosis of VAP and NLR (*r*, 0.220; *P,* 0.010), and between SOFA score and RDW (*r*, 0.446; *P*, 0.000) (Fig. 5a, b).

## Discussion

Pneumonia is the most common nosocomial infection in patients admitted to ICU [45]. Several serum markers expressed when patients exposed to bacterial toxins. Changes in the levels of biomarkers could signal a change in clinical status [46].

This study assessed 136 patients diagnosed as VAP; the mortality rate was 39.7% that was consistent with the American Thoracic Society, and Infectious Diseases Society of America guideline [1] that reported mortality range from 33 to 50%.

In agreement with other studies [47–50], the current study showed a male sex predominance. The age of the study patients was higher in the non-survivors group. Blot et al. and other studies [51, 52] concluded that VAP mortality more with the elderly.

SOFA score helped to predict the severity status and impact of organ failure on the mortality outcome. The admission SOFA score used to assess the degree of organ dysfunction, so it can organize patients into risk categories. While SOFA score taken at time of diagnosis of VAP is prognostic tool. The highest score can diagnose of highest point of multiorgan dysfunction. SOFA was easy to calculate with less data collection. Change in SOFA at the time of diagnosis was a valid tool for the assessment of mortality in different critical illnesses [53–57]. There was a significantly higher SOFA score at diagnosis of VAP in non-survivors in comparison with survivors in this study. These results were consistent with Vincent et al. [58], Ferreira et al. [53], and several other studies [12, 59–61].

In ROC analysis, AUC was 0.81 in the current study, Karakuzu et al. [60] found an AUC 0.821 for SOFA score at the time of VAP diagnosis in mortality predicting. This was also similar to the values obtained in other earlier studies (AUC ranging from 0.72 to 0.89) [60]. Hence, calculating the SOFA score at the time of VAP diagnosis may provide valuable information for mortality prognosis.

Inflammation and oxidative stress affect red cell homeostasis. So RDW showed a strong association with inflammatory biomarkers [13, 62]. The current study showed that higher RDW was statistically significantly associated with increased hospital mortality. This is in accordance with Zhang et al.’s [63] study that was done on critically ill patients mixed ICU of a tertiary teaching hospital. Several studies [28, 64, 65] on patients with sepsis found that RDW considered a clinical importance marker in sepsis management and mortality predication. Lee et al. concluded that RDW was a valuable prognostic marker for mortality in patient with community-acquired pneumonia [62]. In a cohort study done by Chen et al. [66], they found that RDW, albumin level, age, and serum creatinine were independent predictors of mortality in with community-acquired pneumonia. Another retrospective cohort study on pediatric patients reported that the value of RDW on admission was associated with the need for invasive mechanical ventilation and a prognostic parameter of respiratory failure in the pediatric ICU [67].

Neutrophils and other inflammatory cells mediate patients’ pro-inflammatory state in infection [12, 68]. The augmented innate response with neutrophil-mediated killing can suppress apoptosis of neutrophil and thus, neutrophils rising and lymphocytes apoptosis [69, 70]. Recently, the neutrophil/lymphocyte ratio (NLR) has been recorded to mortality prognosis in CAP [42, 71]. Jager et al. [42] study showed elevated NLR in patients with CAP and even higher levels among patients who died in hospital with AUC 0.701. Their study concluded that NLR predicts the severity and outcome of CAP with high prognostic accuracy in comparison with other classic markers of infection.

To the best of our knowledge, few studies have assessed the NLR value in VAP. Feng et al. [12] showed a reasonable performance of high NLR as a prognostic factor of 30-day mortality in VAP. The present study showed good predictive discrimination for mortality (AUC, 0.729). So NLR may help physicians to rapidly classify patients into different prognostic groups, to reduce VAP mortality [12, 72].

The present study has several strengths. To our knowledge, this was the first study that predicts mortality for respiratory patients with VAP by using blood biomarkers either RDW, NLR, or both with good performance. Furthermore, this study found a positive correlation between one of the most important ICU scores (SOFA score) and both blood biomarkers (RDW and NLR). So it suggests that RDW and NLR, which is quickly performed, may act as the scoring systems in determining high-risk patients with VAP.

### Limitation of the study

This study was done in a single tertiary hospital and there may be local differences between centers and institutions.

## Conclusion

NLR and RDW are non-specific inflammatory markers that could be calculated quickly and easily via routine hemogram examination. These markers have comparable prognostic accuracy to severity scores. Consequently, RDW and NLR are simple yet promising markers for ICU physicians in monitoring the clinical course, assessment of organ dysfunction, and predicting mortality in mechanically ventilated patients. Therefore, this study recommends the use of blood biomarkers with the one of the simplest ICU score (SOFA score) in the rapid diagnosis of critical patients as a daily works in ICU.

## Data Availability

The data sets generated and/or analyzed during the present study are not publicly available, but they are available from the corresponding author on reasonable request.

## Abbreviations

VAP: Ventilator-associated pneumonia
RDW: Red cell distribution width
NLR: Neutrophil-lymphocyte ratio
SOFA: Sequential Organ Failure Assessment
ICU: Intensive care units
CBC: Complete blood count
CRP: C-reactive protein level
WBC: White blood cell
MCV: mean corpuscular volume
COPD: Chronic obstructive pulmonary disease
GCS: Glasgow Coma Scale
